# Manufacturing solid-state battery electrodes like an architect

**DOI:** 10.1093/nsr/nwad027

**Published:** 2023-02-03

**Authors:** Yijin Liu

**Affiliations:** Stanford Synchrotron Radiation Lightsource, SLAC National Accelerator Laboratory, USA

Lithium battery is a disruptive technology for energy storage. The adoption of lithium batteries has led to tremendous impacts in industries of consumer electronics and electric vehicles, both of which have demonstrated enormous market value. There is a strong demand for further improving their energy density, fast charging capability, longevity, and safety. The development of solid-state batteries is a potentially viable approach to address these technical challenges and to achieve the performance required to transform the related applications.

Regardless of the chemical compounds used and the cell configurations, the fundamental functioning mechanism of a lithium battery relies on an artificial structure that has the cathode and anode electrodes mixed with, and separated by, a lithium-ion conducting and electron-insulating media, the electrolyte. In the effort to replace conventional liquid electrolytes with solid-state electrolytes, a lot of emphasis has been placed on the intrinsic material properties including the lithium-ion conductivity, electron conductivity, (electro)chemical stability, and mechanical property. The micromorphology, on the other hand, is often regarded as an important factor [1] but has not been extensively investigated. Purposely tailoring the micromorphology could have a very significant impact on the quest for practical solid-state batteries.

Recently, a research group led by Jiajun Wang from Harbin Institute of Technology reported an innovative approach to improve the practical lithium-ion diffusion kinetics in a ∼100-μm-thick solid-state cathode electrode by purposely constructing a structural gradient across its depth (Fig. 1) [2]. Through controlling the spatial variation of the cathode particle size across the electrode, the research team effectively manipulated the tortuosity of the ion transport path as a function of depth. They employed a suite of synchrotron-based experimental tools to characterize the micromorphology across different length scales and the chemical heterogeneity at different electrochemical states. It was found that the designed tortuosity gradient could reduce reaction heterogeneity, suppress cell polarization, and improve capacity retention over prolonged battery cycling.

**Figure 1.**
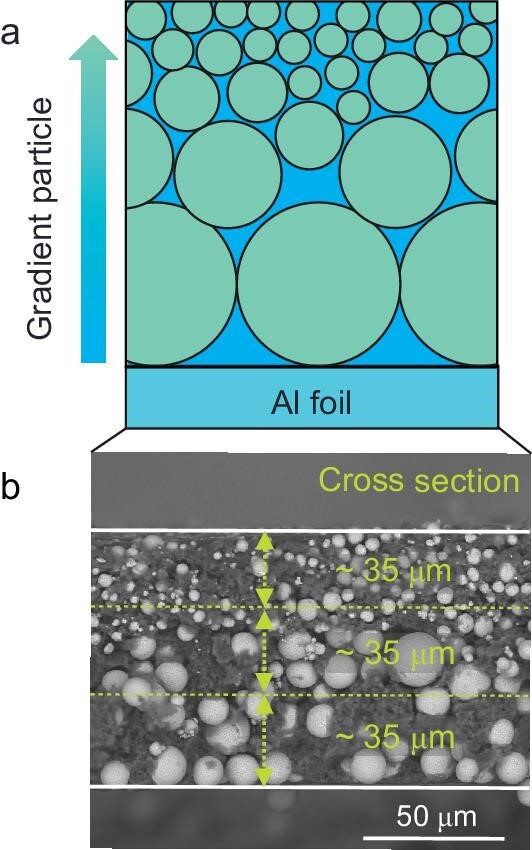
(a) Schematic illustration of the thick cathode electrode with tailored particle size gradient. (b) A scanning electron microscope (SEM) image over the cross section of the electrode. Reproduced with permission from ref. [2].

The detrimental electrochemical polarization effect leads to different degrees of active material utilization at different depths, inducing chemical heterogeneity that could lead to local structural disintegration. It has been suggested that, while morphological consistency is desirable in the lateral (in-plane) direction, the incorporation of a structure gradient along the longitudinal (out-of-plane) direction could be beneficial [3]. The work by Wang *et al.* highlights a structural design strategy that is guided by intuition and is informed by mechanistic understanding of the interplay among stress, charge, and chemomechanical damage in composite battery electrodes [4].

Battery manufacturing requires a delectated control of many processes, which is traditionally optimized through trial-and-error and, thus, is inefficient and expensive. To improve the effectiveness, battery scientists and engineers need to design and manufacture battery components, e.g. solid-state battery electrodes, like a good architect, who not only has the required skills to conduct an artistic design but also understands the practical needs and the underlying fundamentals.

## References

[1] Zhang J , Chen Z, Ai Q et al. Joule 2021; 5: 1845–59. 10.1016/j.joule.2021.05.017

[2] Liu Q-S , An H-W, Wang X-F et al. Natl Sci Rev 2023; 10: nwac272. 10.1093/nsr/nwac272 PMC9977374 36875785

[3] Li J , Sharma N, Jiang Z et al. Science 2022; 376: 517–21. 10.1126/science.abm8962 35482882

[4] Liu P , Xu R, Wang T et al. J Electrochem Soc 2020; 167: 040527. 10.1149/1945-7111/ab78fa

